# Iron(III)–Quercetin Complexes’ Safety for MRI Cell Tracking in Cell Therapy Applications: Cytotoxic and Genotoxic Assessment

**DOI:** 10.3390/nano12162776

**Published:** 2022-08-13

**Authors:** Nathupakorn Dechsupa, Panida Kosintarajit, Kanyapak Kamkan, Thanyalak Khanjina, Chonticha Sirikul, Phattarawadee Innuan, Authaphinya Suwan, Nampeung Anukul, Jiraporn Kantapan

**Affiliations:** 1 Molecular Imaging and Therapy Research Unit, Faculty of Associated Medical Sciences, Department of Radiologic Technology, Chiang Mai University, Chiang Mai 50200, Thailand; 2 Department of Radiologic Technology, Faculty of Associated Medical Sciences, Chiang Mai University, Chiang Mai 50200, Thailand; 3 Center of Radiation Research and Medical Imaging, Faculty of Associated Medical Sciences, Department of Radiologic Technology, Chiang Mai University, Chiang Mai 50200, Thailand; 4 Division of Transfusion Science, Department of Medical Technology, Faculty of Associated Medical Sciences, Chiang Mai University, Chiang Mai 50200, Thailand

**Keywords:** IronQ, theranostics, nanoparticles, genotoxicity, cytotoxicity, regenerative medicine, cell tracking, MRI, PBMCs, ROS

## Abstract

The theranostic agent iron–quercetin complex (IronQ) provides a T1-positive magnetic resonance imaging (MRI) contrast agent. The magnetically IronQ-labeled cells can be used for cell tracking and have active biological applications in promoting cell and tissue regeneration. However, a detailed investigation of IronQ’s cytotoxicity and genotoxicity is necessary. Thus, this study aimed to evaluate the possibility of IronQ inducing cytotoxicity and genotoxicity in peripheral blood mononuclear cells (PBMCs). We evaluated the vitality of cells, the production of reactive oxygen species (ROS), the level of antioxidant enzymes, and the stability of the genetic material in PBMCs treated with IronQ. The results show that IronQ had a negligible impact on toxicological parameters such as ROS production and lipid peroxidation, indicating that it is not harmful. IronQ-labeled PMBCs experienced an insignificant depletion of antioxidant enzyme levels at the highest concentration of IronQ. There is no evident genotoxicity in the magnetically IronQ-labeled PBMCs. The results show that IronQ does not potentiate the cytotoxicity and genotoxicity effects of the labeled PMBCs and might be safe for therapeutic and cell tracking purposes. These results could provide a reference guideline for the toxicological analysis of IronQ in in vivo studies.

## 1. Introduction

Regenerative medicine (RM) is an emerging field of medicine that encompasses a multidisciplinary area of technology development to replace, engineer, or regenerate human cells, tissues, or organs to restore injured tissues or organs and re-establish normal function [1]. Applying nanotechnology to regenerative medicine has become increasingly important in recent decades. The development of nanomaterials that can influence biology allows for more effective regeneration techniques [2]. In recent years, nanotechnology, particularly nanoscale materials in regenerative medicine, has evolved as a growing field of research and has already delivered clinical applications, with nanoscale materials being utilized in drug delivery and as contrast agents for medical imaging [3].

Magnetic nanoparticles (MNPs), especially iron (Fe)-based MNPs, are the most suitable for medical applications due to their unique magnetic properties and customized functionalization [4]. Moreover, iron is a naturally occurring metal in the human body and can be utilized by the body in the subsequent metabolic process when iron-containing nanoparticles are taken up in the body; hence, these MNPs are biocompatible with the human body [5,6]. The example of using MNPs in regenerative medicine comes from applying iron oxide MNPs (IONPs) as a T2 contrast agent in magnetic resonance imaging (MRI) that can enhance the high anatomic contrast, without considering radiation, for an extended period [7,8]. MR imaging is an effective, non-invasive technique for the high-resolution imaging of anatomic tissue and allows in vivo monitoring of the efficacy of cell therapy approaches. MNPs have been used in conjunction with stem cells for various purposes in clinical translational stem cell-based therapies, including tagging, tracking, and activation of the cells [9,10]. Cells can be magnetically labeled with MNPs, allowing non-invasive monitoring of cell migration to the target location, and the internalization of MNPs by cells can positively affect the differentiation and regeneration capacity of the administered cells [11]. Using these techniques optimizes the overall therapy and beneficially increases its therapeutic outcome. However, before MNPs can be considered for use in the clinical practice of regenerative medicine, there is a crucial need to evaluate the toxicity of specific MNPs on the particular cell type of interest [12]. The purpose of such studies is to build a consensus of an ideal labeling agent for stem cell research using MNPs that are nontoxic, validated in their viability and proliferation, while permitting the cells to maintain their differentiation characteristics, thus justifying MNPs’ use in regenerative medicine [13].

We previously developed a novel theranostic agent, also referred to as the iron–quercetin complex (IronQ), consisting of one molecule of iron (Fe^3+^) and two quercetin molecules. IronQ is a potential T1-positive MRI contrast agent, with a longitudinal relaxivity (*r1*) value of 3.70 mM^−1^·s^−1^ in human plasma obtained from a 1.5 Tesla MR machine. The magnetic nanoparticle, IronQ, is spherical in shape and has various sizes. The median size of particles is 81.39 nm, and they are soluble in water. IronQ has a negative charge on its surface and presents a zeta potential of −24.53 ± 1.88 mV. IronQ can be taken up by peripheral blood mononucleated cells (PBMCs) by simple incubation; the accumulation of IronQ in PBMCs depends on the dose and labeling time, which allows for monitoring the IronQ-labeled cells via MRI with the T1-weighted technique. It should be noted that IronQ at 125 µg/mL was safe and could be used in the long-term culturing (21 days) of PBMCs and for sufficiently monitoring the magnetically IronQ-labeled cells via MRI [14]. Surprisingly, the internalization of IronQ at 125 µg/mL into PMBCs can stimulate the differentiation of PBMCs into spindle-shaped cells, which express proangiogenic cell markers and enhance their secreted therapeutic factors, including the angiogenic factor and regenerative tissue factor [15]. Altogether, these results suggest the advantage of the MRI contrast agent, IronQ, which can act as a theranostic agent that can incorporate magnetically labeled IronQ–PBMCs into PBMCs, not only for cell tracking by MRI, but also for the active use of IronQ to stimulate differentiation and promote cell and tissue regeneration. This finding highlights the potential of magnetically IronQ-labeled PBMC cells to develop uses in cell-based therapy for ischemic diseases and chronic wounds.

Nanoparticles’ potential cytotoxicity and genotoxicity are of critical concern and are essential factors in regenerative medicine and tissue engineering [16]. As mentioned earlier, MNPs, as the therapeutic agent in regenerative medicine, require the magnetic labeling of cells with MNPs followed by implantation within the body. Applying the toxic nanoparticle to the body over a long period can significantly negatively affect the therapeutic outcome of the cell-based therapy [17]. Therefore, it is essential to validate the safety of the therapeutic agent before in vivo transplantation, regardless of the clinical efficacy of the technique. To explore the potential therapeutic efficacy of labeled IronQ–PMBCs, we assessed the in vitro cytotoxicity and genotoxicity of the magnetically labeled IronQ–PBMCs through a series of in vitro experiments. The knowledge gained from the in vitro toxicity tests offer a quick and cost-effective method, with minimal ethical concern for gathering preliminary toxicity data, making it possible to move on to in vivo studies.

## 2. Materials and Methods

### 2.1. Chemicals and Reagents

Roswell Park Memorial Institute (RPMI) 1640 medium was purchased from Caisson Lab (Smithfield, UT, USA). Fetal bovine serum, penicillin, and streptomycin were purchased from Gibco (Gibthai, Bangkok, Thailand). Cell Counting Kit-8 assay (CCK-8) was purchased from Abbkine (Wuhan, China). Phytohemagglutinin-L (PHA-L) form was purchased from Merck Millipore (Darmstadt, Germany). 2′,7′-Dichlorofluorescein diacetate (DCHF-DA) and cytochalasin B from *Drechslera dematioidea* were obtained from Sigma-Aldrich (St. Louis, MO, USA). KaryoMAX™ Colcemid™ solution in HBSS was purchased from Thermo Fisher Scientific (Rockford, IL, USA). The iron (III)–quercetin complex (IronQ) was provided by Dr. Nathupakorn Dechsupa, Molecular Imaging and Therapy Research Unit, Faculty of Associated Medical Sciences, Department Radiologic Technology, Chiang Mai University. IronQ is known to have a spherical shape, and the hydrodynamic size of IronQ in Milli-Q water (pH 7.4) is 160.0 ± 2.4 nm. IronQ displays a negative charge on its surface, with the mean zeta potential of −24.53 ± 1.88 mV, which suggests that it was moderately stable in water. The negative zeta potential values imply that IronQ possesses an ionic charge; this characteristic typically prolongs the half-life of the complex in the blood stream by electrostatically repelling plasma proteins. IronQ was dissolved in distilled water to prepare an IronQ stock solution to yield the final concentration of 2 mg/mL. Then, the IronQ solutions were filter-sterilized through a sterile 0.2 µm syringe filter before use.

### 2.2. Peripheral Blood Mononuclear Cells (PBMCs) Isolation and Cell Culture

The peripheral blood mononuclear cells (PBMCs) were collected from healthy human peripheral blood (age 20–40 years, *n* = 8). All donors who participated in this study provided informed consent. The study design was approved by the Human Research Ethics Committee of the Faculty of Associated Medical Sciences, Chiang Mai University (protocol. no. AMSEC-64EX-032, 19 July 2021). The PBMCs were isolated from 50 mL of whole blood from male and female healthy donors using the density gradient centrifugation technique. Briefly, the blood sample was diluted with an equal volume of phosphate buffer saline (PBS, pH 7.4). Then, we gently layered the diluted blood on the top of lymphocyte separation media (Lymphoprep™, Stem cell Technologies, Vancouver, BC, Canada) layer and centrifuged it at 1500 rpm for 30 min at 25 °C to form the white layer of mononuclear cells. Mononucleated cells were collected and cultured in RPMI-1640 medium, with L-glutamine supplemented with 10% fetal bovine serum (FBS) and 1% penicillin/streptomycin at 37 °C in a humidified atmosphere with 5% CO_2_.

### 2.3. IronQ–PBMC Labeling and Morphology Observation

Freshly isolated PBMCs were seeded on 6-well plates at a density of 2 × 10^6^ cells/well in adequate RPMI 1640 culture media, supplemented with 10% FBS. Cells were then treated with or without 125 µg/mL IronQ (IronQ concentrations of 125 µg/mL were used for labeling, as it was previously verified that PBMCs treated with 125 µg/mL enhance the therapeutic potential of PBMCs) [15] and cultured at 37 °C in a humidified atmosphere with 5% CO_2_, without subculture or re-feeding. The change in cell morphology was observed on days 1, 7, and 14 of the incubation periods using an inverted microscope (Nikon, ECLIPSE Ts2, Tokyo, Japan).

### 2.4. Cell Proliferation Assay

The effect of IronQ on cell proliferation was determined using a WST-8 assay kit (Cell Counting Kit-8 (CCK-8) assay). PMBCs (1 × 10^4^ cells/well) in 100 µL of RPMI-1640 medium containing 10% FBS were seeded on 96-well culture plates, treated with different concentrations of IronQ (0–500 µg/mL), and incubated for 1, 3, 7, and 14 days. For the cytotoxic effect of iron ions (Fe^3+^), PBMCs were treated with different concentrations of FeCl_3_ solution (0–500 µg/mL) and further incubated at 37 °C with a 5% CO_2_ supply in a humidified incubator for 72 h. At the indicated time points, 10 µL of CCK-8 solution was added to each well, and the cells were further incubated for 2 h. The absorbance was measured at 450 nm using a microplate reader (BioTek^TM^ Eon^TM^ microplate reader, Winooski, VT, USA). The PBMCs untreated with IronQ were taken as a control. The percent cell viability was calculated as per the formula (mean OD of treated cells/mean OD of control) × 100. 

### 2.5. The Detection of Oxidative Stress in IronQ–PBMC Labeling Cells

The level of reactive oxygen species (ROS) generation in IronQ-labeled PBMCs was determined by DCFH-DA (2′,7′-dichlorodihydrofluorescein diacetate), which acts as a reliable fluorogenic marker for ROS. PBMCs at a seeding density of 1 × 10^6^ per well were seeded in a 24-well culture plate and treated with different concentrations of IronQ (25, 75, 125 µg/mL) for 24 h, and H_2_O_2_ was used as a positive control. To test the effect of iron ions, PBMCs were treated with 200 µg/mL of FeCl_3_ solution for 24 h. At the indicated time, the cells were collected and centrifuged for 1 min at 7000 rpm. The cells were washed two times with PBS, and the cell pellets were incubated in 10 µM of DCFH-DA solution at 37 °C for 30 min. After that, the fluorescence intensity was measured by flow cytometry (Beckman Coulter, Epics XL-MCL, CA, USA). Flow cytometric data were then analyzed by using the FlowJo 10 software (version 10.6, 2019, Becton, Dickinson, NJ, USA).

### 2.6. The Measurement of Lipid Peroxidation in IronQ–PBMC Labeling Cells

Lipid peroxidation (LPO) was determined from the malondialdehyde formation by measuring the colorimetric product (532 nm) formed by the reaction of malondialdehyde (MDA) with thiobarbituric acid (TBA) using the lipid peroxidation assay kit (Sigma-Aldrich, St. Louis, MO, USA), following the manufacturer’s protocol. In brief, IronQ–PBMC labeling cells (2 × 10^6^) were homogenized on ice in 300 µL of the MDA lysis buffer containing 1% butylated hydroxytoluene (BHT) and centrifuged at 13,000× *g* for 10 min to remove insoluble material. In this experiment, 200 µL of the homogenized sample was mixed with 600 µL of TBA and incubated at 95 °C for 60 min to form an MDA-TBA adduct. Then, the mixture was cooled to room temperature in an ice bath for 10 min. After cooling, the optical density of MDA-TBA formation was measured at 532 nm by an Agilent 8453 UV–visible spectrophotometer (Agilent Technologies; Santa Clara, California, CA, USA). The obtained results are expressed in nmol/mL.

### 2.7. The Assessment of Intracellular Antioxidant Status in IronQ–PBMC Labeling Cells

Glutathione (GSH) was measured as per the protocol using a glutathione assay kit (Sigma-Aldrich, St. Louis, MO, USA). The appropriated homogenate sample was added to 5% sulfosalicylic acid to yield a final volume of 10 µL. Then, the mixture solution was mixed with 150 µL of potassium phosphate buffer (95 mM, pH = 7.0) and 0.031 mg/mL DTNB and the mixing solution was incubated at room temperature for 5 min. Then, the reaction was started by adding 0.038 mg/mL NADPH to the mixing solution. The absorbance of the yellow color developed was measured at 412 nm using a microplate reader (BioTek^TM^ Eon^TM^ microplate reader, Winooski, VT, USA). The concentration of GSH was expressed as nmol/mL of the sample.

Catalase (CAT) activity was assessed according to the catalase assay kit (Sigma-Aldrich, St. Louis, MO, USA), following the manufacturer’s instructions. The reaction mixture was obtained by combining 20 µL of the homogenate sample, 30 µL of methanol, and 100 µL of 100 mM phosphate buffer (pH = 7.0). The reactions were then started by adding 20 µL of hydrogen peroxide to the mixed solution and shaking it for 20 min at room temperature. After that, we stopped the reaction by adding 30 µL of potassium hydroxide to each well, added 30 µL of Purpald (Chromogen), and shook the mixture for 10 min at room temperature. The absorbance was measured at 240 nm using a microplate reader (BioTek^TM^ Eon^TM^ microplate reader, Winooski, VT, USA).

Superoxide dismutase (SOD) activity was measured in lysates of PBMCs as per the protocol using the superoxide dismutase (SOD) activity assay kit (Sigma-Aldrich, St. Louis, MO, USA). Briefly, 20 µL of the homogenate sample was added to 160 µL of WST working solution. Then, we started the reaction by adding 20 µL of xanthine oxidase and incubating the plate at 25 °C for 30 min. The change in absorbance was read at 450 nm by the decrease in color development. The SOD activity was expressed as U/mL of the sample.

### 2.8. Genotoxicity Study

Single-cell gel electrophoresis assay (comet assay) was performed according to a method previously described by Singh et al., with slight modifications [18]. A PBMC suspension was mixed with 0.8% low-melting temperature agarose (LMPA) and then placed on pre-coated frosted slides with 1% standard melting temperature agarose. Then, we covered the slide with a coverslip to make a uniform layer and refrigerated it for 5 min. After solidifying, the coverslip was removed, and the prepared slides were then subjected to lysis solution (2.5 M sodium chloride, 10 mM Tris, 100 mM ethylenediaminetetraacetic acid (EDTA), 10% dimethyl sulfoxide, and 1% Triton X-100, pH 10.0), keeping them for at least 2 h. The slides were then incubated in an ice-cold alkaline electrophoresis solution for 20 min, followed by electrophoresis at 15 V for 20 min. After electrophoresis, the slides were neutralized by being immersed in a neutralization buffer for 5 min 3 times. Lastly, the slide was stained by spreading propidium iodide (10 µg/mL) and incubated for 10 min. The slide was then rehydrated in distilled water and visualized under a fluorescent microscope (Nikon, ECLIPSE Ts2, Tokyo, Japan). We manually captured at least 100 nuclei to analyze tail length, tail movement, olive tail movement, and % of DNA in the tail using CometScore software (TriTek Corp., Sumerduck, VA, USA).

A chromosomal aberration assay was performed to evaluate the clastogenic potential of IronQ on PHA-stimulated PBMCs. PBMCs were stimulated by 0.3 mL of PHA in 5 mL of RPMI-1640 medium for 22 h, followed by treatment with different concentrations of IronQ (25, 75, 125 µg/mL) and further incubation for 24 h. The metaphase chromosomes were prepared following Hungerford’s instructions, with some modifications [19]. By adding a 0.01% colchicine solution, the cells were put into a metaphase arresting state and incubated for two hours. PBMCs were then hypotonically treated by being resuspended in 0.075 M KCl and placed in a water bath at 37 °C for 20 min. After that, cells were fixed with Carnoy’s fixative (a 3:1 mixture of methanol and acetic acid) for 20 min. The fixed cells were collected and centrifuged at 300× *g* for 10 min, followed by three-step washing using a fixative solution to resuspend the remaining cell pellets. The prepared slides were made by adding the suspended cells to a slide that had already been pre-chilled and then allowing the slides to air dry. The prepared slides were then stained with 5% Giemsa stain for 10 min. Slides were then observed for metaphase chromosomes under a light microscope (Nikon, Alphaphot YS2, Tokyo, Japan) with a 100× oil immersion lens. Scoring of 50 well-spread metaphase plates was performed per slide. 

Cytokinesis-block micronucleus assay (CBMN) was performed to evaluate the chromosomal breakage in PHA-stimulated PBMCs due to IronQ exposure. The CBMN test was performed according to a method previously described by Schmid et al. [20] and slightly modified by Speit et al. [21]. The PHA-stimulated PBMCs cells were treated with different concentrations of IronQ (25, 75, 125 µg/mL), followed by adding 3.2 µg/mL cytochalasin B to block the cytokinesis 44 h after PHA stimulation, and further incubated for 26 h. At the end of treatment, cells were harvested and treated with ice-cold 0.075 M KCl and centrifuged immediately. The pellet was then fixed three times in Carnoy’s fixative (3:1 methanol/acetic acid), and smears dawned on the pre-coded clean slides and were left to air dry. The slides were then stained with 5% Giemsa stain for 10 min and were observed and analyzed under a light microscope (Nikon, ECLIPSE Ts2, Tokyo, Japan). One thousand binucleated cells were scored, and we recorded the number of micronuclei. The nuclear division index (NDI), determined using the formula NDI = (M1 + 2M2 + 3M3 + 4M4)/N, where M1–M4 represent the proportion of cells with 1–5 nuclei, and N represents the total number of cells scored, was used to assess toxicity.

### 2.9. Statistical Analysis

Data are presented as mean ± standard deviation values (SD). All the experiments were performed on eight independent donors. Statistical analysis was carried out using IBM^®^ SPSS^®^ Statistics Subscription software (IBM Corp. in Armonk, NY, USA). Statistical differences among the treatment groups were assessed by one way ANOVA, followed by Tukey’s multiple comparison post-hoc test. Data were considered statistically significant when a *p*-value less than 0.05 was observed.

## 3. Results

### 3.1. Cytotoxicity Effects Assessment of IronQ Complex in Human Peripheral Blood Mononuclear Cells (PBMCs)

**Figure 1:**
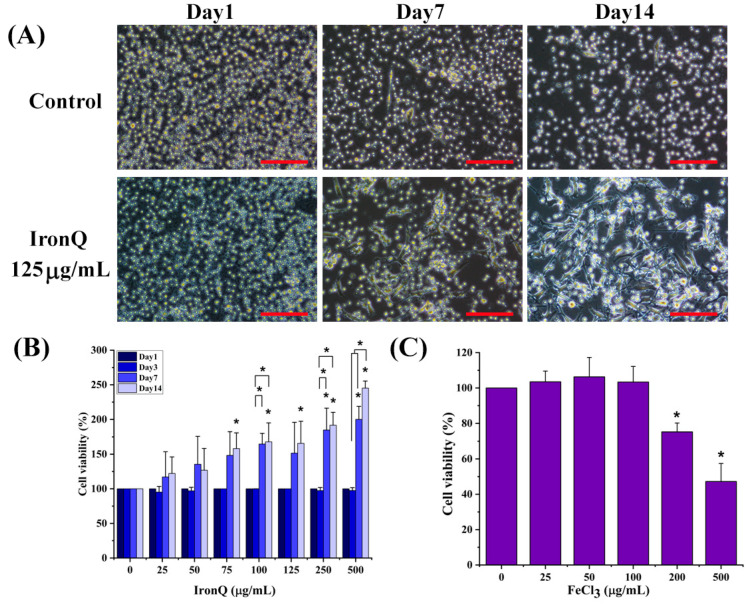
Cytotoxicity effects of IronQ complex in human peripheral blood mononuclear cells (PBMCs). (**A**) Phase-contrast images of the morphological states of PBMC observation at different time points on PBMCs treated with 125 µg/mL of the IronQ. Scale bar = 100 mm. (**B**,**C**) Cellular viability in human PBMCs evaluated by CCK-8 assay after being treated with IronQ nanoparticle and iron ions (FeCl_3_). The data obtained are expressed as mean ± SD (n = 8). * *p <* 0.05, significantly different versus control.

In this study, human peripheral blood mononuclear cells (PBMCs) were used to determine the in vitro toxicity of the IronQ complex because the magnetically labeled IronQ–PBMCs can be used as a therapeutic agent in cell-based therapy for chronic wound healing. Morphological observations at different time points revealed that IronQ stimulates the differentiation of PBMCs by increasing the number of adherent cells, with the unique characteristic of a long spindle cell of considerable length (~100 µm). By increasing the incubation time, PBMCs’ adherence morphology significantly increased and almost reached full confluence on Day 14 (Figure 1A). Cytotoxicity was evaluated by assessing PBMC viability by the Cell Counting Kit-8 (CCK-8) assay after the short and long incubation periods. The results demonstrate that IronQ treatment does not induce any cytotoxic effect in PMBCs, even during a long incubation period with cells. Interestingly, the treatment of IronQ promotes the increasing number of cells in a time- and concentration-dependent manner (Figure 1B). Notably, IronQ-labeled PBMCs did not show decreased viability compared to the unlabeled controls immediately after the labeling procedure or after 72 h in the CKK-8 assay. Moreover, IronQ-labeled PBMCs showed improved proliferation ability over 14 days (Figure 1B). In contrast, cytotoxicity was induced by treating PBMCs with ferric ions (FeCl_3_) at the highest concentration. The viability of PBMCs was reduced to 75% after treatment with 200 µg/mL ferric ions for 72 h and decreased to 47%, detected at the maximal dose of ferric ions at a 500 µg/mL concentration for 72 h (Figure 1C). 

### 3.2. Effect of IronQ-labeled PBMCs on Induction of Intracellular Reactive Oxygen Species (ROS) Generation

**Figure 2:**
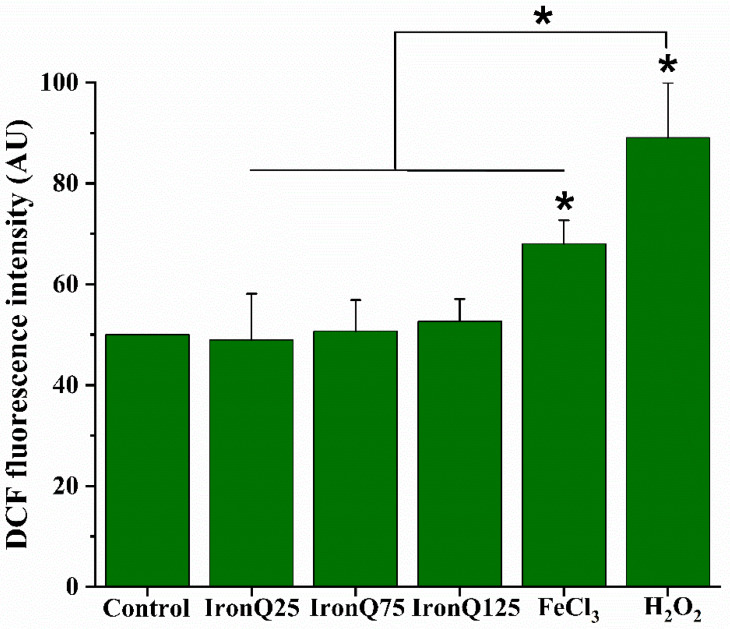
Oxidative stress assessment in human PBMCs upon treatment with various concentrations of IronQ by measuring the ROS level. The bar graph represents the quantitative analysis of ROS formation. PBMCs treated with 500 µΜ H_2_O_2_ were considered as positive controls. Data are expressed as mean ± SD of eight independent samples. * *p* < 0.05, significantly different versus control. ROS: reactive oxygen species; PBMCs: peripheral blood mononuclear cells.

IronQ-labeled PBMCs were investigated for their potential to generate ROS by DCFH-DA staining, followed by flow cytometry. Four doses (0, 25, 75, and 125 µg/mL) of IronQ were tested. The mean fluorescent intensity of DCF of PBMCs treated with IronQ (0, 25, 75, and 125 µg/mL) for 24 h was statistically insignificant compared to the control. At a lower amount of IronQ (25 and 75 µg/mL), no effect on ROS generation was found in PBMCs after IronQ exposure for 24 h. The IronQ complex causes a marginal increase in ROS generation at the highest doses of IronQ (125 µg/mL). However, treating PBMCs with 200 µg/mL of ferric ions (FeCl_3_) for 24 h caused a significant increase in ROS compared to the control. Moreover, after PBMC exposure to 500 µM hydrogen peroxide (H_2_O_2_), which acts as a positive control in this study, a significant increase in the production of intracellular ROS was observed compared to the control PBMCs and other treatments (Figure 2).

### 3.3. Effect of IronQ Complex on the Antioxidant Response and LPO Level in Human Peripheral Blood Mononuclear Cells

**Figure 3:**
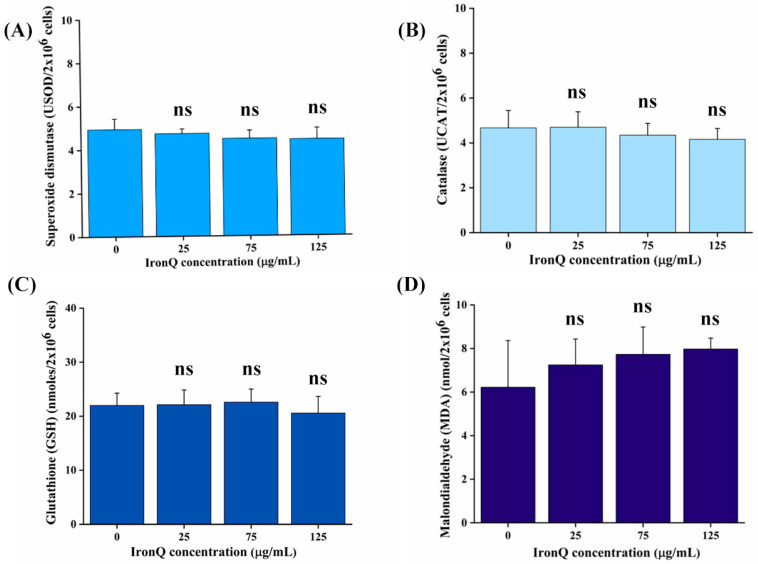
Antioxidant response and the lipid peroxidation level after PBMC labeling with IronQ. (**A**) SOD activity in PBMCs labeled with IronQ at different concentrations for an incubation period of 24 h. (**B**) CAT activity in PBMCs treated with different concentrations of IronQ. (**C**) GSH level in PBMCs after labeling with IronQ at different concentrations for an incubation period of 24 h. (**D**) Malondialdehyde (MDA) level after PBMC labeling with IronQ at different concentrations for an incubation period of 24 h. Data are expressed as mean ± SD of eight independent samples. No significant difference in antioxidant enzyme levels or MDA levels were observed in PBMCs treated with different concentrations of IronQ groups, compared to the control. ns is not significantly different from the control. SOD: superoxide peroxidase; CAT: catalase; GSH: glutathione; PBMCs: peripheral blood mononuclear cells.

Reactive oxygen species (ROS), produced in cells after being exposed to IronQ, and which are harmful to cells, are countered by cellular antioxidant species, such as enzyme systems (SOD, GPx, and catalase) and low-molecular-weight scavengers. The activities of antioxidant enzymes, including superoxide dismutase (SOD) and catalase (CAT), were assessed in PBMCs after labeling with different concentrations of IronQ for 24 h. The levels of SOD and CAT activity in IronQ-labeled PBMCs were found to be marginally decreased compared to the untreated control. SOD and CAT activity decreased in PBMCs with a higher dose (125 µg/mL) compared to the lower dose, but it was also insignificantly lower (Figure 3A,B). The intracellular antioxidant GSH was determined in the control and IronQ-labeled PBMCs. GSH levels in different concentrations of IronQ-labeled PBMCs (0, 25, 75, 125 µg/mL) were marginally lower in the highest dose (125 µg/mL) compared to the control, indicating mild oxidative stress induced by IronQ nanoparticles (Figure 3C). The IronQ, when labeled with PBMCs, showed a slight increase in lipid peroxidation (LPO) with an increase in IronQ concentration, as determined by the MDA level (Figure 3D). However, when compared with the corresponding control group, no significant difference was obtained in the PBMCs labeled with different concentrations of IronQ. The activation of a cellular antioxidant system may have counterbalanced the effect of ROS. The overall results suggest that PBMCs labeled with IronQ at the highest dose (125 µg/mL) are harmless and suitable for cell labeling. 

### 3.4. Genotoxicity Effects of IronQ Complex on PBMCs

#### 3.4.1. Comet Assay 

**Figure 4:**
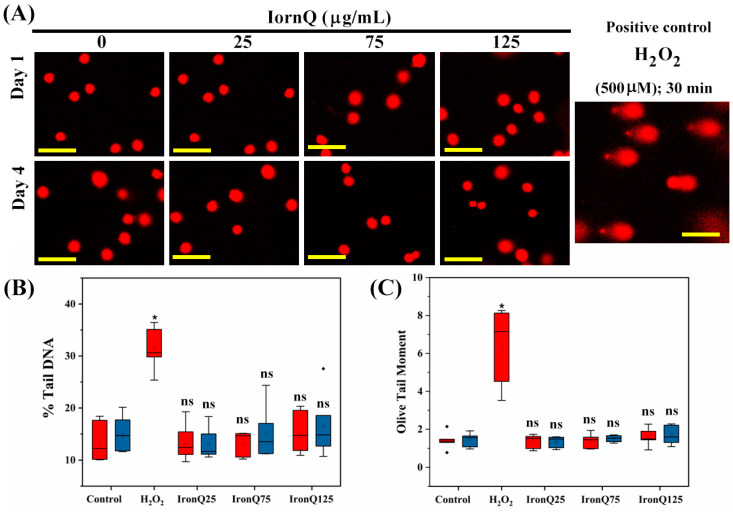
DNA damage assessment of PBMCs after labeling with IronQ by comet assay. (**A**) Representative comet images of PBMCs treated with different concentrations of IronQ for an incubation period of 24 and 96 h. (**B**) Bar diagram showing the assessment in % of DNA in the tail. (**C**) Bar diagram showing the assessment in olive tail moment. Data are expressed as mean ± SD of eight independent samples. A significant increase in DNA damage parameters after PBMC exposure to H_2_O_2_ served as a positive control compared to control PBMCs. However, there was no significant difference in the IronQ treatment groups compared to controls. Asterisks indicate significance (* *p* < 0.05), and ns is not significant, as compared to the control.

Comet assay, or single-cell gel electrophoresis, is a reliable assay to measure genotoxicity, detecting DNA damage in individual cells when treated with any genotoxic agents. The results obtained from the comet assay revealed that there was no evidence of DNA damage induction after labeling PBMCs with IronQ. Figure 4A shows a representative image of a comet obtained after labeling IronQ–PBMCs with varying concentrations for 24 h (Day 1) and 96 h (Day 4). There was no increase in comet parameters, including the % tail DNA and olive tail moment (OTM) values, upon exposure to IronQ (Figure 4B,C). Nevertheless, a slight rise in comet parameters was observed at 125 µg/mL IronQ concentration; however, the different values were not statistically significant compared to the control (Figure 4B). In contrast, a substantial increase in % tail DNA and OTM values was observed after exposure to 500 µM H_2_O_2_ (positive control) compared to the control (Figure 4B,C). The results suggest that either a short (24 h) or long (96 h) time for labeling PBMCs with IronQ did not exhibit genotoxic effects on the DNA of PBMCs.

#### 3.4.2. Chromosomal Aberration Assay

**Figure 5:**
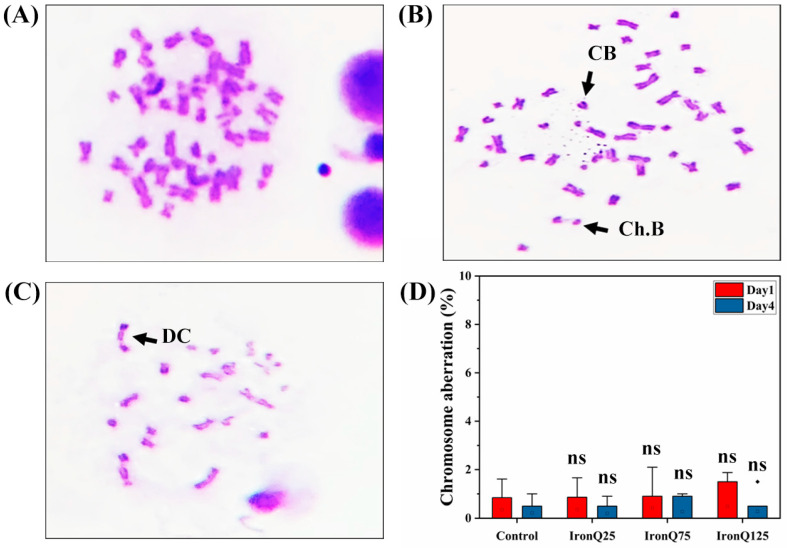
Genotoxicity assessment of PBMCs after labeling with IronQ by chromosomal aberration assay. Representative images showing different chromosome aberrations. (**A**) Normal metaphase chromosome. (**B**) Chromosome breaks and chromatid breaks. (**C**) Dicentric chromosome. (**D**) The graph shows that there was a non-significant increase in chromosomal aberrations on PBMCs labeled with different concentrations of IronQ. Data are expressed as mean ± SD of eight independent samples. ns indicates not significantly different from control. CB: chromatid breaks; Ch. B: chromosome breaks; DC; dicentric chromosome.

The chromosomal aberration (CA) assay is the genotoxic test used to evaluate nanoparticle toxicity at the chromosomal level. For each independent experiment, the numbers of chromosome aberrations were scored on 50 metaphase cells per sample (eight samples/dose). PBMCs labeled with IronQ showed different chromosomal aberrations, including chromosome breaks, chromatid breaks, and dicentric chromosomes. We found that the aberrations with the highest frequency were chromosome and chromatid break, and the aberration with the lowest frequency was a dicentric chromosome (Figure 5A–C). In the PBMCs magnetically labeled with various concentrations of IronQ, the comparative analysis of the numbers of aberrant cells compared with the control showed an insignificant increase in chromosome aberrations when the doses of IronQ increased (Figure 5D).

#### 3.4.3. Cytokinesis-block Micronucleus Assay

**Figure 6:**
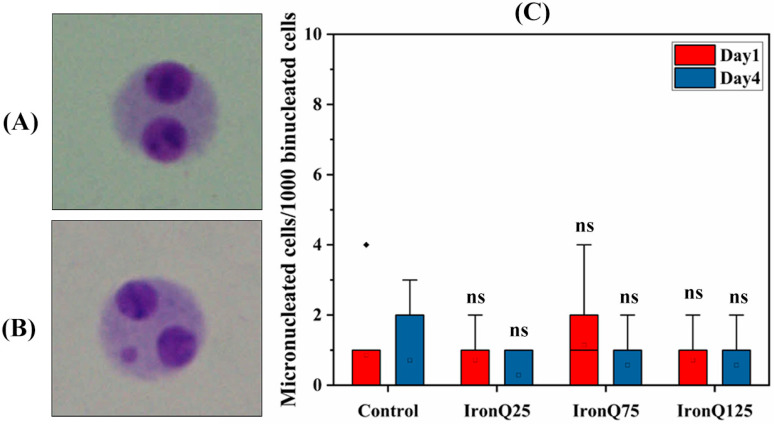
Micronucleus analysis of PBMCs after labeling with IronQ. (**A**) Representative image of PBMCs showing binucleated cells. (**B**) The micronucleus formation among the binucleated cells. (**C**) The graph shows the micronucleus formed among the binucleated cells in PBMCs after labeling with different concentrations of IronQ. Data are expressed as mean ± SD of eight independent samples. The data represent 1000 binucleated cells and ns indicates not significantly different from control.

The genotoxic effects of IronQ on PBMCs were further analyzed by the cytokinesis-block micronucleus assay (CBMN), which visualizes the formation of micronuclei that can be formed during the mitosis of the cell division period, resulting from either chemical exposure-induced chromosome fragmentation or the missegregation of chromosomes. Figure 6A,B show the representative image results obtained from the CBMN assay. PBMCs labeled with various doses of IronQ for 24 h (Day 1) and 96 h (Day 4) showed no evidence of a significant increase in the number of micronucleated cells in a dose-dependent manner compared to the untreated control (Figure 6C). The mean numbers of micronuclei per 1000 binucleated cells were found to be 0.86 ± 1.46 (control), 0.71 ± 0.76 (IronQ 25 µg/mL), 1.14 ± 1.46 (IronQ 75 µg/mL), and 0.71 ± 0.75 (IronQ 125 µg/mL) after 24 h of exposure, and 0.71 ± 1.25 (control), 0.28 ± 0.48 (IronQ 25 µg/mL), 0.57 ± 0.79 (IronQ 75 µg/mL), and 0.57 ± 0.79 (IronQ 125 µg/mL) after 96 h of exposure. The nuclear division index (NDI), a cell proliferation marker in cultures, was calculated and considered a measure of general cytotoxicity (where NDI = 2 indicates that all cells have undergone one division; NDI = 1 indicates no cell division, which occurs if cells with greater chromosomal damage die before cell division). As expected, the cellular division efficacy (NDI) of the IronQ-labeled PBMCs was higher than 1 for all IronQ concentration tests (Table 1), indicating no cytotoxic or cytostatic effects among the IronQ-labeled PMBCs. In addition, the cytokinesis-block micronucleus assay results further corroborated the non-genotoxic effects of IronQ on labeled PMBCs obtained from chromosomal aberration assay and the comet assay. These findings indicate that IronQ-labeled PBMCs did not result in incidences of genotoxicity.

## 4. Discussion

Theranostic nanomedicine is a promising treatment paradigm that is gaining attraction from scientists. In recent years, various nanomaterials, such as theranostic nanoparticles, have emerged as exciting tools in cell-based therapy [10,22]. A theranostic agent is a multifunctional nanoplatform that integrates imaging and therapeutic abilities into one agent [23]. The iron–quercetin complex, also known as IronQ, was recently shown to be a positive contrast for T1-weighted MR imaging. IronQ has high effectiveness in loading into the cells, and a clinical 1.5 T MR scanner visualized the magnetically labeled mononuclear cells. Moreover, it has been shown that IronQ can act as a stimulating agent by favoring the proangiogenic cell differentiation of PBMCs, and the IronQ-labeled PBMCs were still alive for a long time in the culture (21 days) [14]. Pre-conditioning by cultured PBMCs under 125 µg/mL of IronQ enhanced the therapeutic potential of PBMCs, as evidenced by the secreted cytokines and growth factors that support revascularization and tissue repair [15]. Recently, it was reported by our group that mesenchymal stem cells (MSCs) labeled with IronQ do not affect the proliferation and multilineage differentiation capacity of MSCs. Moreover, IronQ-labeled MSCs provide a superior therapeutic effect compared to unlabeled MSCs in the treatment of intracerebral hemorrhage (ICH) in in vivo models. In this work, MSCs labeled with IronQ reduced ICH-induced inflammation, leading to a synergistic role in ameliorating the consequences of ICH, including neurologic deficits, brain edema, and inflammatory response system [24]. These findings suggest that IronQ is highly sensitive and has no toxicity but enhances the therapeutic efficiency of labeled cells. These results indicate that IronQ is the most outstanding theranostic agent in cell-based therapy applications.

Although IronQ has the potential to be used as a theranostic agent in cell-based therapy, no reports on the toxicity of the IronQ complex are available, as IronQ can enter the cells and cause toxicity to the cells. In this study, for the first time, we unravel the cellular event that occurs when IronQ is loaded into the PBMCs. Cell morphology is the first noticeable effect following the exposure of cells to a toxic agent. Microscopic observation of PBMCs treated with IronQ revealed a substantial morphological change, and the treated cells became long, spindle-shaped cells with prolonged incubation time, without any indication of unhealthy cells. It should be noted that nanoparticles have the potential to promote stem cell differentiation and remove several barriers, which will increase their usefulness in regenerative medicine [25,26,27]. The potential effect of IronQ on stimulating the pro-angiogenic differentiation of circulating angiogenic progenitors in peripheral blood was previously reported by our group. It has been shown that 125 µg/mL of IronQ could promote the pro-angiogenic differentiation of mononuclear cells from peripheral blood by increasing the number of early outgrowth colonies (CFU-Hill) on Day 7 after treatment with IronQ and stimulate the transformation of cells to spindle cell phenotypes, characterized as angiogenic progenitor cells though the cell surface markers [15]. The crucial process in toxicology that describes the cellular response to a toxicant is the viability assay, which provides details on cell survival and death. In the present study, we found no toxicity after PBMCs were exposed to various doses of IronQ (0–500 µg/mL), even for a prolonged incubation period (14 days). Inversely, IronQ-treated cells increased the total cell expansion, as evidenced by the gradually increased number of cells after 14 days of incubation of PMBCs with IronQ, while in the untreated control group, the number of cells appears unchanged (Figure 1B). This finding ensures the biocompatibility of IronQ; our results show no evidence of cellular toxicity, and a microscopic study of treated cells shows no indication of cell death, even after a prolonged incubation time. 

The reactive oxygen species (ROS) production after exposure to a nanoparticle, which further causes oxidative stress, is a primary determinant of nanotoxicity [28]. Excessive ROS generation can cause oxidative stress, making it difficult for cells to maintain healthy, redox-regulated functions. This results in DNA damage, genotoxicity, altered cell motility, cytotoxicity, apoptosis, and cancer development [29]. ROS are the byproducts of cellular oxidative metabolism, mainly occurring in the mitochondria. It has been known that ROS play a dual role in cellular biology. Although excessive ROS generation is harmful to cells, it can cause damage to DNA, macromolecules, and organelles and ultimately lead to genotoxicity and cell death. In contrast, ROS at an adequate level is required to trigger critical signaling pathways to promote critical biological processes, including cellular proliferation and differentiation, as ROS play a pivotal role as secondary messengers and influence normal physiological functions [30,31]. It has been reported that the increase in iron in an un-complexed form (not in a protein or other protective metal complex) causes an oxidative imbalance via the stimulation of the production of ROS, mediated mainly by the Fenton reaction; in the presence of metal ions, hydrogen peroxide (H_2_O_2_) receives an electron from metal ions and converts to hydroxyl radicals (^•^OH) [12,32]. Intracellular antioxidant enzymes then counter these free radicals. A study by Huang et al. described that iron oxide nanoparticles could generate ferric (Fe^3+^) ions due to the degradation of iron oxide nanoparticles inside the cells, and these metal ions can cause cytotoxicity to cells [33]. In our study, the effect of 200 µg/mL Fe^3+^ ions, resulting from the dissolution of FeCl_3_, significantly induced ROS generation and cytotoxicity in PMBCs (Figure 1C and Figure 2). In contrast, for IronQ, we found a marginal formation of ROS after labeling with PBMCs, even at the highest concentration (125 µg/mL) used in this study. This result is probably related to the fact that the complexation of ferric (III)–quercetin (IronQ) did not degrade into Fe^3+^ ions, or because the concentration of Fe^3+^ ions after degrading and accumulating in cells is not too high and only causes the quick formation of hydroxyl radicals inside the cells [34].

Redox homeostasis is vital in reducing oxidative damage and stimulating critical signaling pathways. Balancing ROS production is strictly regulated and controlled by effective defensive machinery to prevent excessive ROS production that is harmful to cells [35]. These antioxidant systems consist of enzymatic antioxidants, such as superoxide dismutase (SOD), catalase (CAT), glutathione peroxidase (GPxs), and thioredoxin (Trx), as well as non-enzymatic antioxidants, which collectively reduce the oxidative state [36]. An essential non-enzymatic scavenger cellular defense against oxidative stress is glutathione (GSH). It is well known that cells’ adaptive responses to oxidative stress include changes in their GSH levels. However, any minimal change in GSH levels reveals the cell’s adaptive response to oxidative damage [37]. A considerable decline in GSH levels may be caused by the effective destruction of the ROS produced by severe oxidative stress [38]. IronQ-labeled PBMCs result in only a marginally reduced GSH level at the highest concentration (125 µg/mL) of IronQ used in this study, suggesting that IronQ exposure to cells causes only mild oxidative stress that an intracellular scavenger can counter. Apart from a non-enzyme system, a cellular defense against oxidative stress also uses antioxidant enzymes, such as SOD and CAT, to minimize the production and action of harmful ROS in the system. SOD is the primary defense against oxidative stress from highly reactive superoxide radicals. It catalyzes superoxide radicals to less toxic hydrogen peroxide and molecular oxygen, while GPxs decomposes the more stable hydrogen peroxide at low concentrations and catalase at high concentrations into water [39]. It has been demonstrated that prolonged cellular oxidative stress causes a low SOD level [40]. In the present study, IronQ-labeled PBMCs for 24 h revealed an insignificant alteration in SOD and CAT activity at the highest concentration (125 µg/mL) of IronQ used in this study. A marginal decrease in GSH, SOD, and CAT levels in PBMCs was observed after being labeled with a high concentration of IronQ (75 and 125 µg/mL). The lipid peroxidation (LPO) caused by the excessive ROS degradation of polyunsaturated fatty acids causes damage to the membrane and structural integrity of the cells [41]. In our study, there was an insignificant increase in lipid peroxidation, but the trend seemed to increase dose-dependently after PBMCs were labeled with IronQ (Figure 3D). These findings could be regarded as an early effect of ROS generation in PBMCs. Mild oxidative stress in cells, which the intracellular defense system can readily handle, is the fundamental cause of the non-observation of lipid peroxidation, which means that the cellular defense mechanism can restore this balance. The mild ROS formation in cells may result from the iron ion’s intracellular interactions with cellular machinery.

Metal nanoparticle exposure might be a cause of genotoxicity due to the excessive ROS generation, leading to damage to DNA. In certain situations, it may cause mutagenicity and carcinogenicity [42]. In our study, from the comet assay evaluation of PBMCs treated with various concentrations of IronQ for 24 and 96 h, we did not find any significant increase in DNA damage of PBMCs after incubating with IronQ, even at the high concentration. This suggests that PBMC labeling with IronQ for an extended period does not induce oxidative stress that causes DNA damage. These findings are in concordance with the results of the marginal ROS formation in cells after incubation with IronQ; this mild ROS formation can be handled by the antioxidant response of the cells. The chromosomal aberration, which could be any changes in the number of chromosomes or the structure of the chromosome, is considered a genotoxic marker. The genotoxic agents that induce chromosomal aberrations are believed to be vital markers for cancer risk [43,44]. The IronQ nanoparticles do not cause chromosome break or deletion, which may harm the labeled cells. We observed a non-significant alteration in chromosomal aberration after exposure to IronQ. In addition, the cytokinesis-block micronucleus assay results further corroborated the chromosomal breaks and DNA damage in IronQ-labeled PMBCs. We also found that IronQ-labeled PBMCs did not alter the frequency of micronucleus induction. Our results agree with those of Hlaing et al., who also demonstrated the genotoxic potential of Fe–tannic acid (Fe–TA) nanoparticles in vivo. The authors concluded that Fe–TA NPs do not induce genotoxicity in rats treated with nanoparticles [45]. The IronQ-labeled PBMCs at the highest concentration of 125 µg/mL under the conditions of this study showed marginal ROS formation and a marginal increase in lipid peroxidation. Moreover, we did not find any significant increase in DNA damage, chromosome abnormality, or micronucleus induction. Our findings prove that the IronQ nanoparticles did not induce cytotoxicity or genotoxicity in PBMCs.

Importantly, for MRI-based cell tracking, it is crucial to comprehend the technical proficiency of in vitro stem cell labeling with metal-based contrast agents, regarding its relaxing behavior and the relevance of its biological impacts. Based on this, we previously optimized and reported that 125 µg/mL of IronQ complex containing 10% fetal bovine serum in a short or long incubation period was ideal for effective in vitro labeling of PBMCs. The iron accumulation in PBMCs after incubation with 100 µg/mL of IronQ was approximately 4.54 ± 1.09 pg Fe/cell and 63.29 ± 6.03 pg Fe/cell for an incubation period of 1 and 10 days, respectively. These concentrations are enough for detection by MRI, as it has been reported previously by Heyn et al. that an ideal effective concentration of iron is around 1.4–3.0 pg Fe/cell; thus, our parameters seem to be sufficient for detection with MRI [46]. Based on the toxicity assessment, the previously reported relaxation behavior, and the significance of therapeutic effects, this labeling optimization could suggest the safe and biocompatible properties of IronQ for cell labeling. However, further in vivo toxicity will be required to ensure that PBMC labeling with the IronQ theranostic agent does not induce any harmful effects to the transplant body after implantation. 

## 5. Conclusions

Herein, we have demonstrated the toxicological assessment of PBMCs labeled with IronQ. The studied IronQ concentrations ranged from 0 to 125 µg/mL, the highest concentration (125 µg/mL) being the previously optimized protocol. There was no significant alteration in cytotoxicity or genotoxicity levels after labeling PBMCs with IronQ (125 µg/mL), but we observed increased cell growth. Marginal increases in toxicological parameters, such as ROS generation and lipid peroxidation, were observed after cell labeling, but were non-significant. IronQ-labeled PMBCs experienced an insignificant depletion of GSH, SOD, and CAT levels, which may be due to an early consequence of mild oxidative stress in PBMCs. PBMCs labeled with IronQ (125 µg/mL) for 24 and 96 h demonstrated no apparent signs of genotoxicity. Therefore, labeling PBMCs with the IronQ theranostic agent might be safe for use for therapeutic and cell tracking purposes. These results could provide a reference guideline for the toxicological analysis of IronQ in in vivo studies.

## Figures and Tables

**Table 1:** Micronucleus (MN) formation frequency among the 1000 binucleated cells and the nuclear division index (NDI) in PBMCs after labeling with different concentrations of IronQ (*n* = 8).

Treatment	MN Frequency	NDI
Control (Day 1)	0.86 ± 1.46	1.73 ± 0.03
IronQ 25 µg/mL (Day 1)	0.71 ± 0.76 ^ns^	1.78 ± 0.02 ^ns^
IronQ 75 µg/mL (Day 1)	1.14 ± 1.46 ^ns^	1.92 ± 0.05 ^ns^
IronQ 125 µg/mL (Day 1)	0.71 ± 0.75 ^ns^	1.83 ± 0.04 ^ns^
Control (Day 4)	0.71 ± 1.25	1.66 ± 0.07
IronQ 25 µg/mL (Day 4)	0.28 ± 0.48 ^ns^	1.67 ± 0.01 ^ns^
IronQ 75 µg/mL (Day 4)	0.57 ± 0.79 ^ns^	1.75 ± 0.02 ^ns^
IronQ 125 µg/mL (Day 4)	0.57 ± 0.79 ^ns^	1.69 ± 0.06 ^ns^

The values are expressed as mean ± SD of eight independent samples. Micronucleus formation frequency was determined in 1000 binucleated cells/treatment/sample. NDI was calculated from 1500 cells/treatment/sample. ^ns^ indicates not significantly different from control.

## Data Availability

All data presented in this study are available upon request from the corresponding author.
